# (*E*)-Methyl 2-(3-cinnamoyl­thio­ureido)acetate

**DOI:** 10.1107/S1600536810030084

**Published:** 2010-08-11

**Authors:** Ibrahim N. Hassan, Bohari M. Yamin, Mohammad B. Kassim

**Affiliations:** a School of Chemical Sciences and Food Technology, Faculty of Science and Technology, Universiti Kebangsaan Malaysia, UKM 43600 Bangi Selangor, Malaysia

## Abstract

In the title compound, C_13_H_14_N_2_O_3_S, the methyl 2-(3-formyl­thio­ureido)acetate fragment and the phenyl ring adopt an *E* configuration. The mol­ecule exhibits an intra­molecular N—H⋯O hydrogen bond, which completes a six-membered ring. The crystal packing is stabilized by inter­molecular N—H⋯S contacts, generating a two-dimensional hydrogen-bonding network.

## Related literature

For bond-length data, see: Allen *et al.* (1987 ▶). For related structures, see: Yamin & Hassan (2004 ▶); Hassan *et al.* (2008*a* ▶,*b* ▶,*c* ▶, 2009 ▶); Hung *et al.* (2010 ▶). For the preparation, see: Hassan *et al.* (2008*a* ▶).
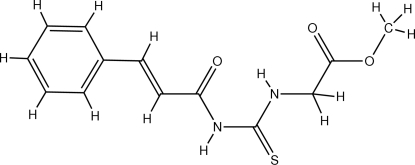


## Experimental

### 

#### Crystal data

C_13_H_14_N_2_O_3_S
*M* *_r_* = 278.33
Triclinic, 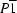

*a* = 4.992 (2) Å
*b* = 11.720 (5) Å
*c* = 12.542 (6) Å
α = 112.999 (7)°
β = 91.492 (7)°
γ = 96.258 (7)°
*V* = 669.6 (5) Å^3^
*Z* = 2
Mo *K*α radiation
μ = 0.25 mm^−1^
*T* = 298 K
0.38 × 0.32 × 0.13 mm

#### Data collection

Bruker SMART APEX CCD area-detector diffractometer
Absorption correction: multi-scan (*SADABS*; Sheldrick, 2000 ▶) *T*_min_ = 0.912, *T*_max_ = 0.969
6562 measured reflections
2466 independent reflections
1689 reflections with *I* > 2σ(*I*)
*R*_int_ = 0.040

#### Refinement

*R*[*F* ^2^ > 2σ(*F* ^2^)] = 0.068
*wR*(*F* ^2^) = 0.173
*S* = 1.12
2466 reflections
180 parameters
2 restraints
H atoms treated by a mixture of independent and constrained refinement
Δρ_max_ = 0.31 e Å^−3^
Δρ_min_ = −0.36 e Å^−3^

### 

Data collection: *SMART* (Bruker, 2000 ▶); cell refinement: *SAINT* (Bruker, 2000 ▶); data reduction: *SAINT*; program(s) used to solve structure: *SHELXS97* (Sheldrick, 2008 ▶); program(s) used to refine structure: *SHELXL97* (Sheldrick, 2008 ▶); molecular graphics: *SHELXTL* (Sheldrick, 2008 ▶); software used to prepare material for publication: *SHELXTL*, *PARST* (Nardelli, 1995 ▶) and *PLATON* (Spek, 2009 ▶).

## Supplementary Material

Crystal structure: contains datablocks global, I. DOI: 10.1107/S1600536810030084/kp2273sup1.cif

Structure factors: contains datablocks I. DOI: 10.1107/S1600536810030084/kp2273Isup2.hkl

Additional supplementary materials:  crystallographic information; 3D view; checkCIF report

## Figures and Tables

**Table 1:** Hydrogen-bond geometry (Å, °)

*D*—H⋯*A*	*D*—H	H⋯*A*	*D*⋯*A*	*D*—H⋯*A*
N2—H2*A*⋯O1	0.86	1.88	2.610 (4)	142
N1—H1*A*⋯S1^i^	0.85	2.61	3.463 (4)	176

Symmetry code: (i) 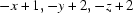.

**Fig. 1.**
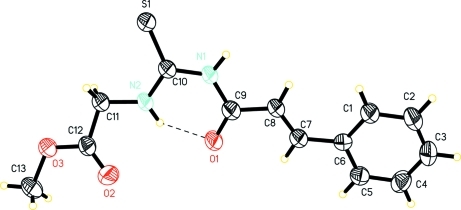
The molecular structure of (I) with displacement ellipsoids drawn at the 50% probability level. Intramolecular hydrogen bond is drawn by a dotted line.

## supplementary crystallographic information

### Comment

The title compound, I, is a methyl ester derivative of glycine thiourea analogoue to our previously reported molecules, methyl-2-(3-benzoylthioureido)acetate (II) (Hassan *et al.*, 2009), ethyl-2-(3-benzoylthioureido)acetate (III) (Hassan *et al.*, 2008*a*), propyl-2-(3-benzoylthioureido)acetate (IV) (Hassan *et al.*, 2008*b*) and butyl-2-(3-benzoylthioureido)acetate (V) (Hassan *et al.*, 2008*c*). Bond lengths and angles in the molecule are in normal ranges (Allen *et al.*, 1987) and comparable to those of the benzoyl derivatives II, III, IV and V. The methyl acetate, [O2/O3/C9/C10/C11] plane is inclined to the phenyl ring, [C1—C6] (A), with a dihedral angle of 26.78 (18)° and this angle is smaller than that of compound (II) [73.4 (2)°]. The phenyl ring and the thiourea fragment, [S1/N1/N2/C9/C10/C11] (B), are essentially planar. In the methyl acetate group, the maximum deviation from the mean plane is 0.037 (2)Å for the atom O3. The dihedral angles of the fragments A/B and B/C are 11.17 (14)° and 20.21 (15)°, respectively, whereas the dihedral angle of the A/C fragments is 73.4 (2)°. There is an intramolecular hydrogen bond, N2—H2A···O1 which completes a six-membered ring (N2/H2A/O1/C9/N1/C10) (Fig. 1) and an intermolecular N1—H1A···S1 hydrogen bond (Table 1) which generates a two-dimensional hydrogen bonding network.

### Experimental

The title compound was synthesised according to a previously reported procedure (Hassan *et al.*, 2008*a*). A colourless crystal, suitable for X-ray structuzre analysis was obtained by a slow evaporation from CH_2_Cl_2_ solution at room temperature (yield 73%).

### Refinement

H atoms of both C and N atoms were positioned geometrically and allowed to ride on their parent atoms, with *U*_iso_=1.2*U*_eq_ (C) for aromatic 0.93 Å, *U*_iso_ = 1.2*U*_eq_ (C) for CH_2_ 0.97 Å, and *U*_iso_ = 1.5*U*_eq_ (C) for CH_3_ 0.96 Å. Hydrogen atoms attached to N were also positioned geometrically and allowed to ride on their parent atoms and with *U*_iso_(H) = 1.2*U*_eq_(N) for N–H 0.86 Å.

### Crystal data

**Table:**

C_13_H_14_N_2_O_3_S	*Z* = 2
*M**_r_* = 278.33	*F*(000) = 292
Triclinic, *P*1	*D*_x_ = 1.381 Mg m^−^^3^
Hall symbol: -P 1	Mo *K*α radiation, λ = 0.71073 Å
*a* = 4.992 (2) Å	Cell parameters from 1753 reflections
*b* = 11.720 (5) Å	θ = 1.8–25.5°
*c* = 12.542 (6) Å	µ = 0.25 mm^−^^1^
α = 112.999 (7)°	*T* = 298 K
β = 91.492 (7)°	Block, colourless
γ = 96.258 (7)°	0.38 × 0.32 × 0.13 mm
*V* = 669.6 (5) Å^3^	

### Data collection

**Table:**

Bruker SMART APEX CCD area-detector diffractometer	2466 independent reflections
Radiation source: fine-focus sealed tube	1689 reflections with *I* > 2σ(*I*)
graphite	*R*_int_ = 0.040
ω scan	θ_max_ = 25.5°, θ_min_ = 1.8°
Absorption correction: multi-scan (*SADABS*; Sheldrick, 2000)	*h* = −6→6
*T*_min_ = 0.912, *T*_max_ = 0.969	*k* = −14→14
6562 measured reflections	*l* = −15→15

### Refinement

**Table:**

Refinement on *F*^2^	Primary atom site location: structure-invariant direct methods
Least-squares matrix: full	Secondary atom site location: difference Fourier map
*R*[*F*^2^ > 2σ(*F*^2^)] = 0.068	Hydrogen site location: inferred from neighbouring sites
*wR*(*F*^2^) = 0.173	H atoms treated by a mixture of independent and constrained refinement
*S* = 1.12	*w* = 1/[σ^2^(*F*_o_^2^) + (0.0832*P*)^2^ + 0.1736*P*] where *P* = (*F*_o_^2^ + 2*F*_c_^2^)/3
2466 reflections	(Δ/σ)_max_ < 0.001
180 parameters	Δρ_max_ = 0.31 e Å^−^^3^
2 restraints	Δρ_min_ = −0.36 e Å^−^^3^

### Special details

**Table:**

Geometry. All e.s.d.'s (except the e.s.d. in the dihedral angle between two l.s. planes) are estimated using the full covariance matrix. The cell e.s.d.'s are taken into account individually in the estimation of e.s.d.'s in distances, angles and torsion angles; correlations between e.s.d.'s in cell parameters are only used when they are defined by crystal symmetry. An approximate (isotropic) treatment of cell e.s.d.'s is used for estimating e.s.d.'s involving l.s. planes.
Refinement. Refinement of *F*^2^ against ALL reflections. The weighted *R*-factor *wR* and goodness of fit *S* are based on *F*^2^, conventional *R*-factors *R* are based on *F*, with *F* set to zero for negative *F*^2^. The threshold expression of *F*^2^ > σ(*F*^2^) is used only for calculating *R*-factors(gt) *etc*. and is not relevant to the choice of reflections for refinement. *R*-factors based on *F*^2^ are statistically about twice as large as those based on *F*, and *R*- factors based on ALL data will be even larger.

### Fractional atomic coordinates and isotropic or equivalent isotropic displacement parameters (Å^2^)

**Table:**

	*x*	*y*	*z*	*U*_iso_*/*U*_eq_	
S1	0.2582 (2)	0.96891 (9)	0.84472 (8)	0.0546 (4)	
O1	0.8785 (5)	1.3017 (3)	0.8950 (2)	0.0691 (9)	
O2	0.6025 (5)	1.2077 (3)	0.5979 (2)	0.0728 (9)	
O3	0.2006 (5)	1.1278 (2)	0.5020 (2)	0.0578 (7)	
N1	0.6772 (6)	1.1430 (3)	0.9392 (2)	0.0423 (7)	
N2	0.4711 (6)	1.1404 (3)	0.7718 (2)	0.0497 (8)	
C1	1.5217 (7)	1.3497 (3)	1.2458 (3)	0.0541 (10)	
H1B	1.4097	1.2766	1.2349	0.065*	
C2	1.7365 (8)	1.3923 (4)	1.3298 (3)	0.0643 (11)	
H2B	1.7663	1.3478	1.3753	0.077*	
C3	1.9055 (8)	1.4992 (4)	1.3466 (3)	0.0596 (10)	
H3A	2.0484	1.5274	1.4035	0.072*	
C4	1.8621 (7)	1.5641 (3)	1.2790 (3)	0.0554 (10)	
H4A	1.9769	1.6362	1.2891	0.066*	
C5	1.6476 (7)	1.5223 (3)	1.1957 (3)	0.0477 (9)	
H5A	1.6196	1.5672	1.1504	0.057*	
C6	1.4733 (6)	1.4153 (3)	1.1780 (3)	0.0400 (8)	
C7	1.2554 (7)	1.3734 (3)	1.0864 (3)	0.0453 (8)	
H7A	1.2414	1.4237	1.0452	0.054*	
C8	1.0742 (6)	1.2719 (3)	1.0546 (3)	0.0428 (8)	
H8A	1.0749	1.2193	1.0942	0.051*	
C9	0.8735 (7)	1.2429 (3)	0.9572 (3)	0.0479 (9)	
C10	0.4759 (6)	1.0896 (3)	0.8501 (3)	0.0394 (8)	
C11	0.2769 (7)	1.0976 (3)	0.6722 (3)	0.0496 (9)	
H11A	0.1038	1.1253	0.6961	0.059*	
H11B	0.2522	1.0070	0.6355	0.059*	
C12	0.3849 (7)	1.1516 (3)	0.5889 (3)	0.0431 (8)	
C13	0.2708 (9)	1.1823 (4)	0.4194 (3)	0.0680 (12)	
H13A	0.1251	1.1594	0.3605	0.102*	
H13B	0.4312	1.1520	0.3841	0.102*	
H13C	0.3027	1.2716	0.4588	0.102*	
H2A	0.591 (6)	1.204 (2)	0.787 (3)	0.076 (14)*	
H1A	0.694 (8)	1.112 (3)	0.990 (3)	0.070 (12)*	

### Atomic displacement parameters (Å^2^)

**Table:**

	*U*^11^	*U*^22^	*U*^33^	*U*^12^	*U*^13^	*U*^23^
S1	0.0615 (6)	0.0558 (6)	0.0520 (6)	−0.0166 (4)	−0.0175 (4)	0.0350 (5)
O1	0.0748 (18)	0.0797 (19)	0.0662 (17)	−0.0313 (15)	−0.0313 (14)	0.0561 (16)
O2	0.0507 (17)	0.109 (2)	0.0733 (19)	−0.0241 (16)	−0.0172 (14)	0.0620 (18)
O3	0.0570 (15)	0.0750 (18)	0.0525 (15)	−0.0159 (13)	−0.0175 (12)	0.0450 (14)
N1	0.0475 (16)	0.0468 (17)	0.0386 (16)	−0.0049 (13)	−0.0107 (13)	0.0270 (14)
N2	0.0589 (19)	0.0551 (19)	0.0407 (16)	−0.0160 (15)	−0.0173 (14)	0.0324 (15)
C1	0.054 (2)	0.058 (2)	0.058 (2)	−0.0086 (18)	−0.0137 (18)	0.0368 (19)
C2	0.066 (3)	0.077 (3)	0.061 (2)	−0.005 (2)	−0.019 (2)	0.046 (2)
C3	0.053 (2)	0.070 (3)	0.050 (2)	−0.004 (2)	−0.0173 (18)	0.023 (2)
C4	0.050 (2)	0.048 (2)	0.061 (2)	−0.0087 (17)	−0.0087 (18)	0.0193 (19)
C5	0.051 (2)	0.047 (2)	0.051 (2)	−0.0028 (17)	−0.0041 (17)	0.0278 (18)
C6	0.0392 (18)	0.0427 (19)	0.0408 (18)	−0.0029 (15)	0.0001 (15)	0.0217 (16)
C7	0.047 (2)	0.049 (2)	0.0449 (19)	−0.0022 (17)	−0.0054 (16)	0.0267 (17)
C8	0.0451 (19)	0.050 (2)	0.0400 (19)	−0.0011 (16)	−0.0061 (15)	0.0271 (16)
C9	0.053 (2)	0.050 (2)	0.045 (2)	−0.0034 (17)	−0.0062 (17)	0.0270 (18)
C10	0.0424 (19)	0.0425 (19)	0.0379 (18)	0.0021 (15)	−0.0024 (15)	0.0221 (16)
C11	0.051 (2)	0.059 (2)	0.045 (2)	−0.0092 (18)	−0.0124 (17)	0.0330 (17)
C12	0.046 (2)	0.048 (2)	0.0401 (19)	0.0011 (17)	−0.0079 (16)	0.0246 (17)
C13	0.083 (3)	0.083 (3)	0.056 (2)	−0.009 (2)	−0.012 (2)	0.052 (2)

### Geometric parameters (Å, °)

**Table:**

S1—C10	1.666 (3)	C3—H3A	0.9300
O1—C9	1.226 (4)	C4—C5	1.380 (5)
O2—C12	1.186 (4)	C4—H4A	0.9300
O3—C12	1.329 (4)	C5—C6	1.383 (4)
O3—C13	1.445 (4)	C5—H5A	0.9300
N1—C10	1.381 (4)	C6—C7	1.456 (5)
N1—C9	1.383 (4)	C7—C8	1.330 (5)
N1—H1A	0.859 (10)	C7—H7A	0.9300
N2—C10	1.333 (4)	C8—C9	1.466 (5)
N2—C11	1.447 (4)	C8—H8A	0.9300
N2—H2A	0.859 (10)	C11—C12	1.502 (4)
C1—C6	1.383 (5)	C11—H11A	0.9700
C1—C2	1.387 (5)	C11—H11B	0.9700
C1—H1B	0.9300	C13—H13A	0.9600
C2—C3	1.371 (6)	C13—H13B	0.9600
C2—H2B	0.9300	C13—H13C	0.9600
C3—C4	1.370 (5)		
			
C12—O3—C13	116.2 (3)	C8—C7—H7A	116.1
C10—N1—C9	127.9 (3)	C6—C7—H7A	116.1
C10—N1—H1A	120 (3)	C7—C8—C9	120.0 (3)
C9—N1—H1A	112 (3)	C7—C8—H8A	120.0
C10—N2—C11	124.3 (3)	C9—C8—H8A	120.0
C10—N2—H2A	114 (3)	O1—C9—N1	121.8 (3)
C11—N2—H2A	122 (3)	O1—C9—C8	122.9 (3)
C6—C1—C2	120.3 (3)	N1—C9—C8	115.3 (3)
C6—C1—H1B	119.8	N2—C10—N1	116.0 (3)
C2—C1—H1B	119.8	N2—C10—S1	123.9 (2)
C3—C2—C1	120.8 (4)	N1—C10—S1	120.2 (2)
C3—C2—H2B	119.6	N2—C11—C12	107.6 (3)
C1—C2—H2B	119.6	N2—C11—H11A	110.2
C4—C3—C2	119.4 (3)	C12—C11—H11A	110.2
C4—C3—H3A	120.3	N2—C11—H11B	110.2
C2—C3—H3A	120.3	C12—C11—H11B	110.2
C3—C4—C5	119.8 (3)	H11A—C11—H11B	108.5
C3—C4—H4A	120.1	O2—C12—O3	124.0 (3)
C5—C4—H4A	120.1	O2—C12—C11	125.7 (3)
C4—C5—C6	121.7 (3)	O3—C12—C11	110.3 (3)
C4—C5—H5A	119.2	O3—C13—H13A	109.5
C6—C5—H5A	119.2	O3—C13—H13B	109.5
C1—C6—C5	117.9 (3)	H13A—C13—H13B	109.5
C1—C6—C7	122.8 (3)	O3—C13—H13C	109.5
C5—C6—C7	119.2 (3)	H13A—C13—H13C	109.5
C8—C7—C6	127.8 (3)	H13B—C13—H13C	109.5

### Hydrogen-bond geometry (Å, °)

**Table:**

*D*—H···*A*	*D*—H	H···*A*	*D*···*A*	*D*—H···*A*
N2—H2A···O1	0.86	1.88	2.610 (4)	142
N1—H1A···S1^i^	0.85	2.61	3.463 (4)	176

Symmetry codes:  (i) −*x*+1, −*y*+2, −*z*+2.
